# Hearing Pronouns Primes Speakers to Use Pronouns

**DOI:** 10.1162/opmi_a_00178

**Published:** 2025-01-04

**Authors:** Jennifer E. Arnold

**Affiliations:** Department of Psychology and Neuroscience, The University of North Carolina at Chapel Hill, Chapel Hill, NC, USA

**Keywords:** priming, language production, reference form, pronouns

## Abstract

Speaking requires frequent decisions about how to refer, for example whether to use a pronoun (she) or a name (Ana). It is well known that this choice is guided by the discourse context, but little is known about the representations that are activated. We use priming to test whether this choice can be facilitated through recent exposure, and if so, what representations are activated. In a storytelling task, participants take turns with experimenters telling a story that is illustrated in 2-panel cartoons. The first sentence is given, and participants describe the second panel in their own words. We manipulate whether the experimenter used a pronoun or name in the prior story. Experiment 1 provides the first evidence in the literature that reference form choice can be primed, and that it is not dependent on the syntactic position of the antecedent. However, the effect is not finely tuned to the preceding prime. Instead, exposure at the start of the experiment persists throughout, even when the prime changes. Experiments 2 and 3 further show that exposure to pronoun primes result in greater pronoun use than at baseline, but that there is no sensitivity to the prime on the most recent trial. Results argue against a role for production facilitation in pronoun use, which suggests that reference production is not impacted by production efficiency.

## INTRODUCTION

Speaking requires frequent decisions about how to refer, for example whether to use a pronoun (*she*) or a name (*Ana*). This choice is constrained, for example pronouns are more natural for recently-mentioned characters (*Ana arrived and she …*) than previously unintroduced characters (e.g., Chafe, 1976). But the choice is not grammatically determined, and speakers instead choose a form based on the context. In the last several decades, researchers have identified numerous features of the discourse context that are associated with the selection of pronouns and other under-specified expressions (e.g., Ariel, 1990; Chafe, 1976; Gundel et al., 1993; Stevenson et al., 1994; Tily & Piantadosi, 2009; see Arnold & Zerkle, 2019 for a review). But less is known about the cognitive mechanisms driving reference form selection, and specifically which representations are activated during this process. Here we tackle this question by asking whether pronoun production can be primed by recent exposure to pronouns, and if so, what types of representations support pronoun use.

It is well established that priming affects choices during language production. That is, speakers are more likely to use words and structures they have recently encountered. For example, activating a semantic representation leads to automatic and partial activation of multiple words (Damian & Bowers, 2003; Dell, 1986; Levelt et al., 1999; Schriefers et al., 1990). When an unintended word receives enough activation, it can be erroneously produced, or can lead to a slow-down in the production of the target word. This shows us that language production involves the activation of concepts (in the message representation) before the activation of the words, and that representations compete for activation. At the sentence level, speakers tend to re-use abstract structures they recently heard (e.g., Bock, 1986; Pickering & Branigan, 1998). This demonstrates that people activate abstract representations and store them in memory.

Priming research reveals two properties of the language production system. First, it identifies the building blocks of speech planning. For example, syntactic priming is excellent evidence for the psychological reality of syntactic structures (Bock & Griffin, 2000; Chang et al., 2006; Ziegler & Snedeker, 2018, see Pickering & Ferreira, 2008 for a review and Mahowald et al., 2016 for a meta-analysis). Second, priming effects are one example of how language behavior changes as a result of exposure to input. At least for the short term, there is a bias to re-use activated structures, which affects the structures that are most frequent in the language and thus available to comprehenders (MacDonald, 2013). It has also been hypothesized that priming leads to implicit learning, which shapes the architecture of the processing system over time (Chang et al., 2006).

Yet much less is known about the key discourse representations that are activated during language production. Here we specifically focus on the production choice between pronouns and more specific referring forms. What representations are activated? Does recent exposure change referential form choices?

Perhaps surprisingly, no previous experimental work has asked whether priming affects the speaker’s choice between referential forms, for example between pronouns and names. It is well established that pronouns are favored in certain discourse contexts, for example when referring to something that was recently mentioned or that appeared in a prominent discourse position such as grammatical subject (e.g., Ariel, 1990; Arnold, 1998; Chafe, 1976; Gundel et al., 1993; Stevenson et al., 1994). Pronouns are also more common when there is only one person in the context than two (Arnold & Griffin, 2007). Thus, scholars agree that speakers must choose their expressions to be appropriate for the discourse context. But no-one has tested whether this choice can be primed.

We do know that pronouns are produced more quickly if the speaker had just produced a pronoun to refer to the same referent, but this evidence comes from a task where speakers were instructed when to use a pronoun (Jescheniak & Schriefers, 1999). We also know that for modified noun phrases, the structure of the modification can be primed (e.g., *the shiny star* vs. *the star that is shiny*; Cleland & Pickering, 2003) but this does not involve a choice between different levels of referential specificity.

In related work, corpus studies show evidence for a phenomenon known as “persistence”: that is, speakers tend to repeat referential forms within a discourse segment, especially for reference to the same entity (e.g., Cameron & Flores-Ferrán, 2004; McKee et al., 2011; Torres Cacoullos & Travis, 2014; Travis, 2007). For example, Travis (2007) examined corpora of conversations in Spanish from New Mexico and Colombia. She examined variation in the use of null pronouns (e.g., ø como; *ø eat-first person*) vs. overt pronouns (e.g., yo como; *I eat*). Variation between these forms is influenced by numerous discourse properties, including discourse status, where overt pronouns are more common for less-prominent or contrastive references (Carminati, 2002; Medina Fetterman et al., 2022). In addition, Travis demonstrated that speakers tended to re-use expressions when referring to the same person over a short span of utterances.

Corpus work thus suggests that priming may guide speakers’ choices in reference form. However, these patterns in naturalistic data can be explained by multiple features that tend to be confounded. One problem is that many of these studies confound priming and discourse status (e.g., Flores-Ferrán, 2002, as reported by Cameron & Flores-Ferrán, 2004; Travis, 2007). For example, McKee et al. (2011) examined variation between null and expressed referring expressions in two sign languages. They found that the strongest predictor of a null form was repeated mention in subject position from one clause to the next. They interpreted this as structural priming. However, this effect could also be explained in terms of the discourse status of the referent, where reduced forms are preferred for reference to the subject or topic of the previous clause while descriptions/names are preferred for non-subjects or less-recently mentioned material (e.g., Brennan, 1995; Gundel et al., 1993), and the use of a particular form (e.g., a pronoun) can impact the perceived discourse status of the referent (Kameyama, 1996). Indeed, in Torres Cacoullos and Travis’s (2014) analysis of variation in the expression of *I* vs. null (in English), subject continuity is highly confounded with the priming effect.

Another challenge with corpus analysis is that the content of the discourse may modulate stylistic choices, such as whether to use more formal or less formal forms (for an effect of formality variation see Callaghan & Travis, 2021), or variation in personal speech styles. Corpus analyses typically take each token as an independent datapoint, but speakers’ choices may be correlated for many reasons. Thus, it remains to be shown whether recent exposure or production of a particular referential form can impact subsequent decisions about what form to use at an abstract level.

Our approach to studying pronoun priming seeks to integrate two different theoretical traditions: 1) mechanistic language production models and 2) research on the discourse conditions that impact reference form choices. In the mechanistic tradition, researchers have sought to understand the representations that are activated during language production. Models of language production suggest that speakers activate a nonlinguistic message representation, which then activates the relevant words and structures, and at the final stage activates the necessary sound sequences. (e.g., Bock, 1986; Dell, 1986; Fromkin, 1971; Garrett, 1975; Levelt, 1989).

Within the mechanistic tradition, pronoun production has mostly been studied as a grammatical agreement problem, in particular in languages like German where words are gender-marked (e.g., Jescheniak et al., 2001). This problem emerges after a speaker has decided to use a pronoun. By contrast, our question is how speakers decide which form to use in the first place. Hypotheses about how this selection takes place could stem from work on the selection of near-synonyms (e.g., sofa vs. couch). Jescheniak and Schriefers (1998) found that multiple words are activated before one near-synonym is selected for production, suggesting that reference forms may also compete during selection.

By contrast, the discourse tradition for studying pronoun production has aimed to identify the conditions under which speakers select one form or another. Within this tradition, Arnold and Zerkle (2019) argue that there are two broad approaches. On the one hand, “pragmatic selection accounts” have found that referential forms tend to vary from more-reduced (e.g., pronouns, especially acoustically reduced pronouns) to more explicit (names or descriptions, or even modified expressions like “the red book”; Ariel, 1990; Chafe, 1976; Gundel et al., 1993), and that these forms systematically correlate with the discourse status of the referent. When the referent is conceptually salient or accessible in the context, people tend to use reduced expressions, but when it is less accessible, they tend to use explicit expressions like names or pronouns. Arnold (2016) proposed that even though these studies don’t explicitly define a mechanism, they imply that there must be a mechanism of selection, e.g., “if referent is salient then pick pronoun”. The closest proposal to this comes from Schmitt et al. (1999), whose model includes a gating mechanism whereby discourse focus selects for pronouns over descriptions, but without any discussion of what defines discourse focus. This view is also implied by Ariel’s notion that referential expressions are “accessibility markers”, which means that they provide a signal to the comprehender about the accessibility status of the referent in their mental model.^1^

A second approach comes from rational accounts (e.g., Orita et al., 2015; Tily & Piantadosi, 2009), which suggest that word selection emerges from two constraints: predictability and the need for successful communication on the one hand, and the speakers’ need for efficient production mechanisms on the other. When a referent is already highly predictable in context, the listener does not need as much explicit input to identify the referent. This account capitalizes on the fact that discourse status is correlated with referential predictability (i.e., the likelihood that a referent will be mentioned again). For example, recently-mentioned things are statistically more likely to be re-mentioned than any particular other thing (Arnold, 1998), and semantic constraints can also affect how likely it is that an entity will be re-mentioned (e.g., Arnold, 1998; Kehler et al., 2008; Stevenson et al., 1994). When referents are predictable, the speaker is justified in using a less-specific form to meet the goal of production efficiency.

Although the selectional and rational accounts are not identical (and many open questions remain), they both essentially capture the effect of the discourse context on referential choice. The purpose of the current work is to turn instead to questions about how this discourse context guides the activation of abstract categories, and whether abstract activations modulate the speaker’s choice of referential form. The answer to this question is important in its own right, and additionally has the potential to shed light on models of reference production. Specifically we ask three questions.

First, does discourse context entirely account for referential choice? Here we are primarily concerned with the choice between more- and less-explicit expressions, a choice that is exemplified by the use of pronouns vs. names/descriptions in English. Selectional accounts do not say anything explicit about retrieval mechanisms, but they seem to imply that the discourse context is the only (or at least the major) consideration. If so, there is no reason to think that hearing or reading pronouns per se would encourage speakers to use pronouns more often, unless the same referent is involved. If person A uses a pronoun for a particular referent, e.g., “Ana went for a walk in the park last weekend. At one point she fell down”, the pronoun itself contributes to the discourse status of Ana, making it more salient or accessible. If person B refers again to Ana in the same discourse, they are likely to also use a pronoun, e.g., “Yeah, I heard that she broke her leg.” However, the question on the table here is not whether hearing a pronoun affects discourse status. Rather, the question is whether hearing a pronoun increases the activation of pronouns generally (or some related representation), such that speakers are more likely to use pronouns for other referents. Thus, if person B instead introduces a new story, e.g., “Liz had to study for an exam. She/Liz stayed up all night,” we want to know if they would be more likely to choose a pronoun for anaphoric reference instead of a repeated name, given that they recently heard a pronoun for a different referent. If we find evidence of abstract priming of referential forms at all, it would show that the production of referential forms is guided by more than just discourse status.

Rational accounts by contrast predict that production is driven by both predictability (which is a correlate of discourse status) and efficiency. Efficiency could be interpreted as a measure of production processing ease, for example Tily and Piantadosi (2009) suggest “Intuitively, the advantage of using pronouns is that they are short and perhaps also easier to produce for other reasons” (see also Orita et al., 2015). This could imply that if other factors modulate the ease of producing one form or the other, it should favor that form. Thus, this account might predict priming pronouns should make producing a pronoun easier and thus increase the rate of choosing this form.

Second, if priming occurs, what representations are primed? This is relevant because people could encode reference at many levels of specificity. Consider “Liz had to study … She …”. This sequence could be stored at a very specific level, e.g., as an instance of a subject pronoun with an antecedent that also occurred in subject position. If so, hearing this might encourage subsequent use of pronouns for referents that occurred in subject position but not other positions. Alternatively priming could rely on representations at the word level, such that the activation of specific words (“she”) would prime the use of the same word, but not other pronouns (“he”). Or people could instead encode pronouns at a more general level, for example the class of anaphoric pronouns, such that “he” primes the production of “she” and vice versa.

Third, is priming short-lived or long-lasting? Pickering and Branigan (1998) proposed that syntactic priming can be explained by a residual activation mechanism, where hearing or producing a structure results in the activation of both the words and structures involved. Such activation is automatic and makes the structure more available during the production of subsequent utterances. While it’s not clear how long such activation could last, it seems likely that it should decay fairly quickly. In support of this mechanism, results show that priming is strengthened if key words are repeated, in the so-called “lexical boost” effect (Pickering & Branigan, 1998; Scheepers et al., 2017). The residual activation model seems consistent with rational models, which propose that pronouns are produced in part because they are easy. If so, increasing the activation of the words or structures involved in production should make them even easier to produce, but this effect should be short-lived.

Alternatively, Chang et al. (2006) proposed that syntactic priming reflects implicit learning, where exposure to unexpected input molds the system over time. This mechanism is consistent with evidence for longer-lasting priming effects. For example, Bock and Griffin (2000) found that primed syntactic structures remained active after 10 trials. If reference form priming results from implicit learning, the effects could be long-lasting.

If reference form priming occurs, another possibility is that it results from a situation-specific shift in the accessibility of a particular discourse style. Pronouns are used as a cohesion device and help signal that two utterances are connected. By contrast, explicit expressions like names or descriptions are appropriate in contexts when the speaker is aiming for clarity and success in referent identification. Thus, exposure to pronouns may signal that the appropriate style for the current situation is one where co-reference is signaled through anaphoric forms like pronouns, or other reduced forms. This view predicts that priming should affect the entire episode and not just the immediately following utterances.

A critical feature of this project is that it tests whether referential form is primed within a discourse context and with a design that carefully controls the discourse features that are known to affect referential form choice. As described above, most variance in referential form choices is driven by the discourse context. Here we manipulate whether the context includes one or two people, which has strong effects on pronoun use (Arnold & Griffin, 2007). We also control for the syntactic position of the antecedent, which is important since speakers tend to use pronouns more for subjects than objects/obliques (Rohde & Kehler, 2014; Stevenson et al., 1994).

To test these questions, we conducted three storytelling experiments conducted in a one-on-one zoom session. Our biggest question was whether we would observe any priming of referential form choice. The cleanest example of this would be a case where speakers mimic the forms used by their interlocutor. We know that syntactic priming occurs both for forms repeated by the same speaker (e.g., Bock, 1986) and different speakers (Branigan, 2007; Branigan et al., 2000; Cleland & Pickering, 2003). We also know that there is substantial inter-speaker variation in pronoun use within experimental tasks, which may stem from differences in expectations about how to do the task (e.g., Zerkle & Arnold, 2016, 2019). Thus, we expect natural differences in the rate of pronoun use across speakers, which would complicate the analysis of any priming effects. In addition, it is difficult to control the participants’ use of pronouns unless we explicitly instruct them, which draws attention to the manipulation. Thus, we asked whether participants would follow the forms used by their conversational partner. In order to simplify the protocol we simply asked experimenters to perform the task with participants.

## EXPERIMENT 1A AND 1B

Experiment 1 was designed to test three questions. First, does priming affect referential form at all? We asked participants to help tell stories in a task where they took turns with the experimenter. The experimenter’s stories provided the primes prior to the participant’s target trials. Second, does priming (if it occurs) depend on whether the prime and target follow the same structure? In Experiment 1a they did (the antecedent was in the same position for both), and in Experiment 1b they did not (the antecedent was in second position for the primes but in first position for the targets). Third, does priming guide only the immediately following trials, or do its effects persist? To test this, we presented primes in blocks, such that half the participants saw only pronoun primes for the first block of the experiment, and the other half saw only name primes for the first block; in the second block the prime type reversed. If priming persists, we should see effects of the first block carrying over to responses in the second block, even though the prime type changes.

Our blocked priming design additionally offers the best opportunity to test whether priming has any effect on referential form at all. Given that this is the first foray into this area, we started with a design that is likely to elicit the biggest effect. If priming has a small effect, it may be strengthened by hearing multiple primes of the same type within a block (Branigan, 2007). If we do see an effect, we can probe its limits in subsequent studies.

Experiments 1a and 1b were initially run as separate experiments; the analyses are combined here for simplicity, with estimates of the priming effects for each experiment separately. For this and all experiments in this study, we aimed for roughly 60 participants per experiment. As this was the very first experimental test of referential priming in production we had no basis for a power analysis; we estimated that a sample size of 60 would be sufficient given that this far exceeds the sample sizes used in other dialogue-based priming experiments (Branigan, 2007; Branigan et al., 2000; Cleland & Pickering, 2003).

### Methods

#### Participants.

120 students participated in exchange for course credit; 60 in Experiment 1a (ages 18–23 (*M* = 19; 39 female, 20 male, 1 nonbinary) and 60 in Experiment 1b (ages 18–43, *M* = 19.6; 38 female, 17 male, 5 nonbinary). 2 additional participants in Experiment 1a were replaced for not meeting our criteria for native speaker status.^2^

#### Materials and Design.

**Figure 1.**
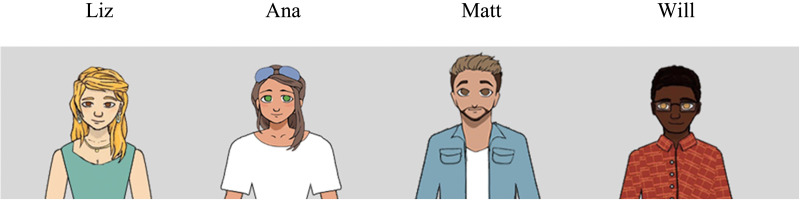
Participants learn characters’ names and pronouns before the experiment.

Participants were asked to help tell stories on the basis of pictured cartoons. All stories depicted four characters: Liz (she), Ana (she), Matt (he), and Will (he). During the instruction period, the experimenter introduced the character names and their pronouns with the picture in Figure 1, and then the participant was tested on their memory for the names and pronouns of each character. If they got any wrong they were corrected and asked to re-do the test.

**Figure 2.**
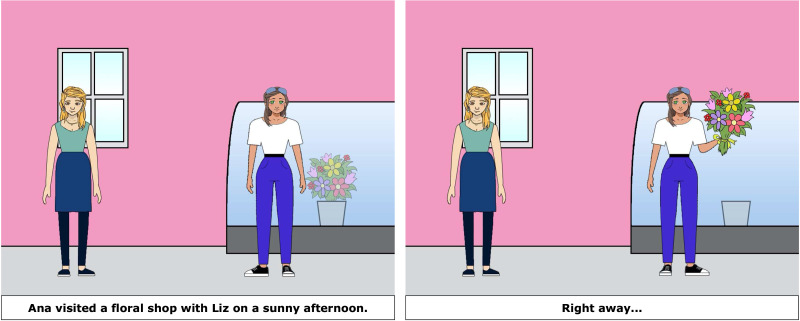
Example Experimenter Priming Item: Experimenter reads first panel and either says “She bought a bouquet of flowers” or “Ana bought a bouquet of flowers.”

**Figure 3.**
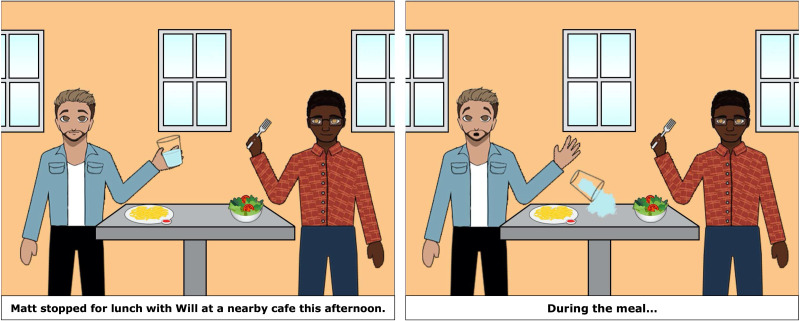
Example Participant Critical Item: Participant reads first sentence and prompt, and finishes the story based on the picture, e.g., “he spilled some water.”

During the main task, the participant and experimenter took turns telling stories based on two-picture sequences, as shown in Figures 2 and 3. Both experimenter and participant viewed the pictures together on a shared zoom screen. The first panel included a written sentence, (e.g., *Matt stopped for lunch with Will at a nearby café this afternoon*), and the second panel included a prompt phrase (e.g., *During the meal …*). The pictures and stories were adaptations of those created for Arnold et al. (2024). Participants first read the context sentence aloud, then the experimenter pressed a button to display the second panel. The participant then read the prompt aloud and continued the second sentence based on the scene depicted in the second panel. Experimenter trials worked the same way except that the experimenter’s continuation was scripted, unbeknownst to the participant.

There were 12 critical trials that the participant completed. These stories were manipulated to include either one or two characters. In both conditions the target character was doing an activity in the first panel, and then a new action in the second panel. In the two-character condition, a second character was introduced with a with-phrase and was present in the picture but not doing any special action in the second panel. In both one and two-person trials, the target character was mentioned as the subject of the first sentence. In Experiment 1a the number of characters was manipulated within items; in Experiment 1b it was manipulated between items.

Each participant trial was preceded by an experimenter trial, where the experimenter read the prompt and provided an ending to the second sentence. For the 12 critical trials, the experimenter trial represented the priming manipulation. Critically, the continuation was scripted such that it included either a name or pronoun for the target character. The prime followed a similar structure as the critical trial, and the second sentence referred to one person doing something. For Experiment 1a, the prime trial always followed the same structure as the critical trial (that is, it had either one or two people, and the target was mentioned as the subject of the first sentence). For Experiment 1b, the prime trial always had two people, and the target was always the second person in the context sentence. Thus, in Experiment 1a the prime had the same pronoun-antecedent structure as the target, and in Experiment 1b it had a different pronoun-antecedent structure (see Table 1). In both experiments, the prime trial included characters of the opposite gender than the following target trial, so any priming effect cannot be specific to the word itself (*he* vs. *she*). In the two-person context, the critical participant trials always pictured both people in the second panel, but some of the prime trials only pictured the target person in the second panel.

**Table 1.** Example primes and critical stimuli for Experiments 1a and 1b.

Experiment 1a prime, one person condition. Ana visited a floral shop on a sunny afternoon. Right away {Ana/she} bought a bouquet of flowers.
Experiment 1a prime, two person condition. Ana visited a floral shop with Liz on a sunny afternoon. Right away {Ana/she} bought a bouquet of flowers.
Experiment 1b prime. Liz visited a floral shop with Ana on a sunny afternoon. Right away {Ana/she} bought a bouquet of flowers.

Target trial, one person condition. Matt stopped for lunch at a nearby café this afternoon. During the meal … [picture shows Matt spilling water]
Target trial, two person condition. Matt stopped for lunch with Will at a nearby café this afternoon. During the meal … [picture shows Matt spilling water]

We also considered the possibility that priming may have long-term effects on production routines as opposed to flexibly modulating responses on a trial-by-trial basis. To test this, we presented the primes in blocks. Thus, each participant saw 6 priming trials in one condition (all name or all pronoun), and then another 6 in the other condition. Thus, the experiment design (including both Experiments 1a and 1b) was 2 (one vs. two people) × 2 (name vs. pronoun prime) × 2 (pronoun prime block first vs. second) × 2 (same structure (Experiment 1a) vs. different structure (Experiment 1b) prime).

In addition to the 12 critical trials and 12 priming trials, there were 14 fillers (7 participant and 7 experimenter trials), which had a different structure. There were also two practice items. The experimenter did the first practice (*Matt and Ana went to a party last night. At the party, Ana danced under the disco ball*.). The participant did the second practice (*Liz and Will went to the grocery store before Thanksgiving. Right away …. [picture of Will picking up a turkey]*). Neither practice story supported the use pronouns in the second sentence. These trials were combined into four lists in Experiment 1a (where the one/two manipulation was within items) and into two lists in Experiment 1b (where the one/two manipulation was between items). For all linguistic stimuli see supplementary materials.

#### Procedure.

The experiment was conducted over zoom in a one-on-one session with the experimenter. Participants read and signed a consent form through Qualtrics, and then completed a demographic survey on Qualtrics. The experimenter then asked the participant to turn off their video, and turned off their own video. The participant was instructed to replace their zoom name with their participant number to maintain anonymity of the recording. The experimenter then shared their screen and launched a powerpoint document with the instructions and stimuli. The experimenter introduced the characters, tested the participant on their names and pronouns, and provided instructions for the task. The participant had a chance to answer questions before starting the task. There were two practice items; on these items the experimenter corrected any name or pronoun mistakes, but did not correct any mistakes during the main task. The entire task typically lasted around 15 minutes.

### Analysis

Research assistants listened to the audio recording of the experimental session. They transcribed the practice items and the 12 critical trials; they also listened to the experimenter’s priming trials and coded whether they were delivered as intended.

For the critical trials, RAs coded whether the participant read the context sentence and prompt accurately. Trials were excluded from analysis if they changed the meaning or structure of the context sentence, but were not excluded for errors on the prompt or for minor errors or disfluencies. They also coded the word used to refer to the target character (Ana, Liz, Matt, Will, she, he), and whether it was a name or pronoun. Trials were also excluded if they repaired the referring expression, used the wrong name, or responded in a way that did not put the target character in the subject position of the response. 86 trials (6%) were excluded (31 for Experiment 1a; 55 for Experiment 1b) for the following reasons: the subject of the sentence was not the target or was plural, missing, or ambiguous (*n* = 59), wrong name or repaired name/pronoun (*n* = 25), a different sentence structure (*n* = 2).

### Results and Discussion

**Figure 4.**
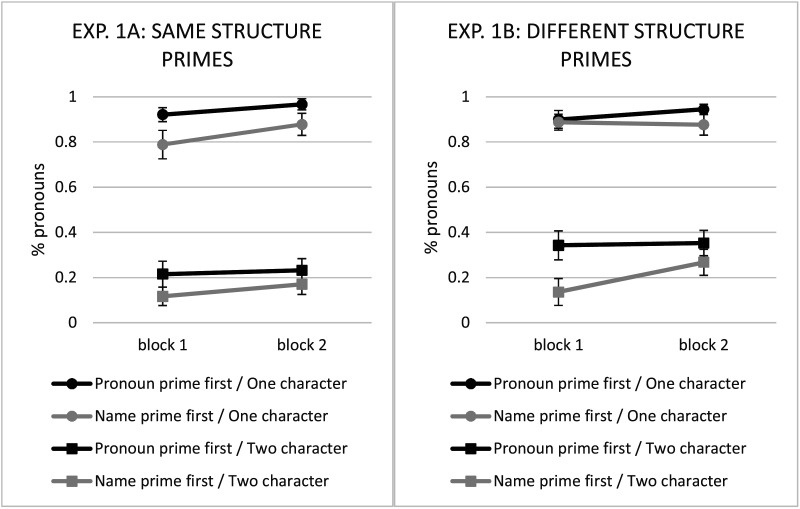
Experiment 1, pronoun use by condition. Left panel: Experiment 1a (same structure primes); Right panel: Experiment 1b (different structure primes). Error bars are standard error of subject means.

Results (see Figure 4) revealed that participants produced more pronouns when they encountered the pronoun prime block first than when they encountered the name prime block first—that is, the black lines are higher than the grey lines. However, there was no effect of the current prime block—that is, the difference between the black and grey lines is generally consistent for the first block (left side) and second block (right side). Unsurprisingly, people also produced more pronouns for one-character than two-character trials (circle-marked lines are higher than square-marked lines).

We tested these effects in a mixed effects logistic regression with SAS proc glimmix, using a binomial distribution and a logit link. We included all manipulated effects (One vs. two characters; current prime; first block prime) and their interactions, plus the effect of prime structure (Experiment 1a vs. Experiment 1b) and two-way interactions between it and the manipulated predictors. We had maximal random effects (random intercepts for participants and items, and random slopes as appropriate).

These findings (Table 2) revealed that pronoun use can indeed be primed: hearing pronoun primes in the first block resulted in greater pronoun use over the experiment. However, this effect was not fine-tuned to the most recently encountered prime trials. The priming effect for the first block carried over to the second block even though the experimenter primes changed. It was also not fine-tuned to the type of antecedent heard in the prime: there was no difference between Experiments 1a and 1b, even though the primes in Experiment 1b all had nonsubject antecedents and the targets all had subject antecedents. The immediately preceding prime always had a different gender from the target trial, although the persistent effect of priming means that it is impossible to tell whether priming is due to re-use of the same word or the general pronoun class. Both “he” and “she” were used as primes in the first few trials, so they could have each independently boosted use of pronouns throughout the experiment.

**Table 2.** Inferential statistics for Experiments 1a and 1b.^3^

	**Est. (*SE*)**	***t***	***p***
**Intercept**	0.62 (0.21)	2.92	0.008
**One vs. Two characters**	2.2 (0.14)	16.19	<.0001
**Current prime (Pronoun vs. Name prime in current block)**	0.03 (0.11)	0.23	0.821
**First-block prime (Pronoun vs. Name prime in first block)**	0.46 (0.17)	2.65	0.013
**Same structure (Experiment 1a) vs. Diff structure (Experiment 1b) prime**	−0.2 (0.17)	−1.22	0.231
**One/Two char × Current prime**	−0.12 (0.11)	−1.07	0.32
**Current prime × First-block prime**	−0.23 (0.17)	−1.4	0.191
**One/Two char × First-block prime**	0.06 (0.12)	0.48	0.639
**First-block prime × Structure**	0.03 (0.17)	0.19	0.851
**Pronoun prime × Structure**	0 (0.11)	0.04	0.97
**One/Two char × Structure**	0.15 (0.12)	1.25	0.212
**One/two char × Current prime × First-block prime**	−0.12 (0.12)	−0.98	0.359

Thus, this experiment revealed that priming does indeed affect reference form choices. However, the priming effect was small. When we tested the effect of first-block prime in each experiment separately with estimates, it narrowly failed to reach significance (see Table 3). We therefore sought to replicate the priming effect in an experiment that manipulates prime type between participants, and with a stronger manipulation of the prime.

**Table 3.** Estimates of the first-block prime effect for Experiment 1a and Experiment 1b.

	**Est. (*SE*)**	***t***	***p***
**Experiment 1a Pro vs. Name prime first effect**	0.99 (0.49)	2.02	0.052
**Experiment 1b Pro vs. Name prime first effect**	0.86 (0.49)	1.74	0.092

## EXPERIMENT 2

To replicate the priming effect of Experiment 1, we used a between-subjects priming manipulation, only using different-structure primes. Thus, if priming occurs again, it could not be due to the repeated antecedent structure. Following Pickering and Branigan (1998), we used a sequence of two experimenter primes preceding each critical trial, which was expected to strengthen the priming effect through cumulative exposure. In addition, we tested the baseline rate of pronoun production prior to the first prime.

### Methods

#### Participants.

69 students participated in exchange for course credit. One was excluded because the experimenter inadvertently gave feedback about the referential form. Our analysis included 68 participants (ages 18–22; *M* = 19; 42 female, 26 male, 1 female/nonbinary); 35 participants in the name-prime condition and 33 in the pronoun-prime condition.

#### Materials and Design.

The task and design were the same as Experiment 1, except for the following differences. First, there were two experimenter primes preceding each of the 12 critical items. 12 of these were identical to the primes in Experiment 1, and 12 new ones were constructed. All the prime and target trials had two characters. The trial immediately preceding the critical trial included two same-gender participants (just like the critical trial). In order to create variation across the items, the prime trial before that included two different-gender participants. While no-one has tested the role of same- vs. different-gender contexts in reference form priming, we do not predict that it should matter in that the effect of gender match on pronoun use is fairly small (Rosa & Arnold, 2017), and no model suggests a fundamentally different process depending on the gender context. Both prime trials involved a reference to the second-mentioned person, which was manipulated to be a pronoun or name. The target character depicted in the critical trial was always the subject of the preceding sentence, whereas the critical name or pronoun in the prime trials always referred to the second character from the first sentence, thus using a different structure (like Experiment 1b). On 8 trials the target character was the same on the preceding prime and target trials, but the stories were different and the first-mentioned character was different. On 4 trials the target and preceding prime were different. Table 4 displays an example sequence. Prime type was manipulated between subjects, such that list 1 only included name primes and list 2 only included pronoun primes.

**Table 4.** Example primes and critical stimuli for Experiment 2.

Experimenter prime #1: Ana ate ice cream with Will today at the creamery. At one point, {Will/he} dropped his ice cream.
Experimenter prime #2: Liz visited a floral shop with Ana on a sunny afternoon. Right away {Ana/she} bought a bouquet of flowers.
Critical trial: Matt stopped for lunch with Will at a nearby cafe this afternoon. During the meal … [picture shows Matt spilling water]

Each critical trial was followed by a participant-response filler trial using a different structure; this sequence was preceded by two prime trials. Thus, the experiment consisted of the experimenter and participant taking turns to complete the stories, where each person did two stories at a time. There were 48 trials in total, plus 4 practice trials. These were divided in two lists that were identical except for the name or pronoun used in the prime trials.

Another difference from Experiment 1 was that the practice trials were designed to be used as a baseline measure of pronoun use prior to hearing the primes (Table 5). There were first two experimenter practice trials. Both of these used a different structure than the critical trials; one involved a name reference to a previously unmentioned character, and one used “they” to refer to two characters previously mentioned in a conjoined NP (Ana and Will). The two participant practice trials had the same structure as the critical trials, with a context sentence introducing two characters in the structure X did something with Y. The second picture depicted the first-mentioned person doing something interesting while the other person was unchanged. The first practice included two same-gender characters (like the critical trials), whereas the second practice included two different-gender characters.

**Table 5.** Practice/Baseline trials used for both lists.

1. Experimenter: Matt sang the vocals for the new recording. Then Liz played the drums.
2. Experimenter: Ana and Will threw a birthday party last night. After that they washed the dishes.
3. Participant: Will talked with Matt all morning at a coffee shop. At one point … [picture shows Will eating a sandwich].
4. Participant: Liz ran a race with Will for the school track team. During the race [picture shows Liz winning].

#### Procedure.

The procedure was the same as for Experiment 1.

### Analysis

The same approach to coding and analysis was used as for Experiment 1. 79 trials were excluded because the participant used an incorrect name and/or corrected the name (*n* = 16), or because the grammatical subject of the response was not the target character (*n* = 63). 8% of the post-priming trials and 16% of the baseline trials were excluded, leaving 873 trials in the final analysis.

### Results and Discussion

**Figure 5.**
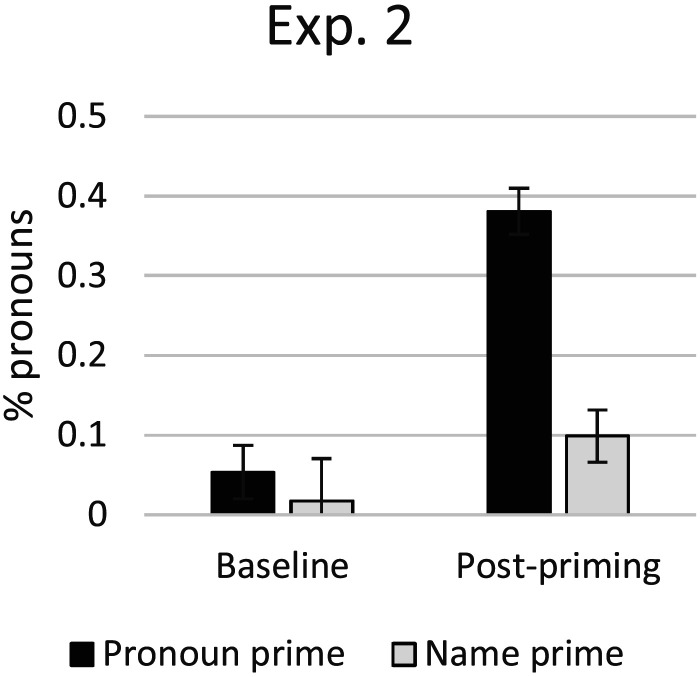
Experiment 2, pronoun use by condition. Error bars are standard error of subject means.

As shown in Figure 5, participants used very few pronouns at all during the baseline trials. For participants on the pronoun-prime list, a total of 3 out of 56 trials used a pronoun (5%), whereas on the name-prime list, a total of 1 out of 58 trials used a pronoun (2%).

By contrast, on the post-priming trials, participants were more likely to produce pronouns following the pronoun prime than following the name prime (see Figure 5). On the pronoun prime list, 4/33 (12%) of the participants produced no pronouns at all, only using names. By contrast, on the name prime list, 22/35 (63%) of the participants produced no pronouns at all. Even if we only consider participants who produced one or more pronoun, the rate of pronouns for the post-priming trials was higher on the pronoun-prime list (43%) than on the name-prime list (27%).

A logistic regression confirmed that these data exhibit a main effect of pronoun priming, as well as a main effect of the contrast between baseline and post-priming trials (see Table 6). Even though the interaction was not significant, planned simple effects were conducted with estimates (see Table 7), which confirmed that the prime effect was significant for the post-priming trials and not for the baseline trials.

**Table 6.** Inferential statistics for Experiment 2.^4^

	**Est. (*SE*)**	***t***	***p***
**Intercept**	−2.72 (0.4)	−6.78	<.0001
**Pronoun prime vs. Name prime**	0.79 (0.36)	2.2	0.029
**Post-priming vs. Baseline**	0.94 (0.39)	2.38	0.02
**Post-priming × Pronoun prime**	0.32 (0.35)	0.93	0.356

**Table 7.** Estimates for the pronoun prime effect for baseline and post-prime trials.

	**Est. (*SE*)**	***t***	***p***
**Pronoun prime effect for post-prime trials**	2.22 (0.5)	4.48	<.0001
**Pronoun prime effect for baseline trials**	0.93 (1.32)	0.7	0.485

We additionally tested whether the priming effect was any greater for the critical trials where the target and preceding prime character were repeated. We found it was not: the difference in pronoun use between the pronoun and name prime lists was 29% for the repeated character trials, and 27% for the not-repeated character trials. When we added repetition to a model of critical trials only, the effect of priming remained significant (*b* = 1.12 (*SE* = 0.26); *p* < .0001) but there was no effect of character repetition (*b* = −0.38 (*SE* = 0.41); *p* = 0.38) nor an interaction with the priming effect (*b* = −0.003 (*SE* = 0.30); *p* = 0.99). This is not surprising given that each trial represented a completely new story, so there was no expected carry-over of the discourse status of characters in the previous story.

These results replicate the finding from Experiment 1, where exposure to pronoun primes increases the rate of pronoun production. For participants who heard name primes, there was very little change in their pronoun use from baseline (2%) to the post-priming trials (10%). For participants who heard pronoun primes, there was a dramatic increase in pronoun use from baseline (5%) to the post-priming trials (38%).

These findings replicate the situation-wide priming effect observed in Experiment 1. We manipulated priming type between lists, so participants were never exposed to the opposite prime type. This is consistent with the idea that referential form priming is long-lasting. However, an open question is whether participants may additionally exhibit a response to the immediately preceding item. If priming occurs via automatic activation of pronoun lexical items or the pronoun class, we might expect that activation to be especially strong immediately following the prime. Experiment 3 tested this prediction.

## EXPERIMENT 3

To test whether pronoun priming can be modulated on a trial-by-trial basis, we repeated Experiment 2 except that the primes alternated within lists and not just between lists. Our question was whether pronoun production would follow the most recently encountered primes.

### Methods

#### Participants.

62 students participated in exchange for course credit. One was excluded due to technical problems with the recording, leaving 61 in the analysis (ages 18–44, *M* = 19.9; 44 female, 14 male, 2 nonbinary).

#### Materials, Design and Procedure.

Experiment 3 used the same procedure, stimuli and list structure as Experiment 2, with two changes. First, the pronoun and name prime conditions were manipulated within each list, and not between lists. Second, we sought a stronger baseline measure of pronoun use by increasing the number of participant baseline trials to 4 (in addition to 4 experimenter baseline trials). In addition, the baseline trials were presented simply as the initial trials in the main task. Prior to the main task, the experimenter and participant each did one practice item (Experimenter: Ana went to the party with Matt last night. At the party they danced [image shows both dancing^5^]; Participant: Will went with Liz to the grocery store before Thanksgiving. Right away … [image shows Will buying a turkey]).^6^

#### Analytical Strategy.

The same procedure for coding and analysis was used as for Experiments 1 and 2, with the exception that we performed two analyses: 1) a comparison between the baseline trials and all main-task trials (both pronoun and name primes together), and 2) a comparison between the pronoun and name prime conditions for the main task.

Of the total possible 976 trials (244 baseline; 732 critical trials), 122 (13%) were excluded because the subject of the response was not the target character (*n* = 112) or the referring expression was incorrect or repaired (*n* = 8). There were 854 trials included in the analysis.

### Results and Discussion

**Figure 6.**
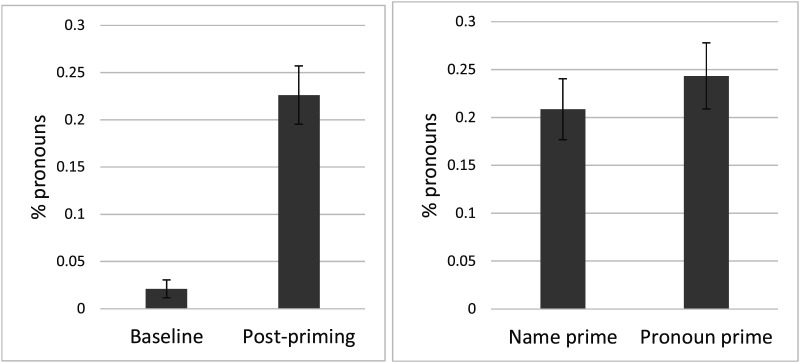
Experiment 3: pronoun use by condition. Left panel shows baseline vs. post-priming (critical) trials. Right panel shows the critical trials divided by name and pronoun prime conditions.

As in Experiment 2, the rate of pronoun use at baseline was very low (4 out of 190, or 2%), but pronoun use rose to 23% during the critical trials. However, there was no difference between trials that immediately followed a name prime (21%) and those that immediately followed a pronoun prime (24%). See Figure 6 for results. Our statistical analysis confirms these patterns: there was a main effect of the baseline vs. critical trials comparison (Table 8a). However, in an analysis of just the critical items (Table 8b), there was no effect of pronoun vs. name prime.

**Table 8a.** Inferential statistics comparing baseline to critical items.^7^

	**Est. (*SE*)**	***t***	***p***
**Intercept**	−2.85 (0.33)	−8.59	<.0001
**Post-priming vs. Baseline**	1.26 (0.36)	3.54	0.0007

**Table 8b.** Inferential statistics comparing name prime to pronoun prime trials for the critical items only.^8^

	**Est. (*SE*)**	***t***	***p***
**Intercept**	−1.63 (0.28)	−5.77	<.0001
**Pronoun vs. Name prime**	0.1 (0.13)	0.8	0.4604

The failure to find a priming effect does not appear to be due to low power. Although we did not do a power analysis prior to the study, we performed a post-hoc power simulation using SimR in R (Green & MacLeod, 2016), based on findings from the critical trials from Experiment 2. These were identical to the critical trials in Experiment 3 except that the priming manipulation was between subjects. We simulated 2000 datasets, and used powerCurve to test the percentage on which the priming effect was significant for different numbers of participants (up to 80) in a logistic regression model with glmer that included Pronoun Prime as a predictor, random effects for subjects and items, and a random slope for pronoun prime by items. This simulation estimated that 90% power would be achieved with 30 participants, suggesting that our sample size of 61 participants is sufficient for replicating the effect. The fact that we didn’t underscores the finding that the effect disappears with a within-subjects manipulation.

The low rate of pronoun use on the baseline trials is consistent with findings from Experiment 2. This suggests that in this task, participants are unlikely to use pronouns if they haven’t heard a pronoun used by the experimenter. This pattern is supported by an examination of the first two post-priming trials. On list 1, the rate of pronoun use was 0% for the first post-priming trial (name prime condition) and 27% for the second post-priming (pronoun prime condition). On list 2, the rate of pronoun use was 21% for the first post-priming trial (pronoun prime condition) and 25% for the second post-priming trial (name prime condition). This suggests that hearing one pronoun prime trial is enough to trigger the strategy of using pronouns, but participants do not respond to the immediately preceding trial in a fine-grained manner.

Again we tested whether character repetition affected the rate of priming, and it did not. The difference in pronoun use between the pronoun-prime and name-prime trials was 11% for the nonrepeated character trials and 8% for the repeated character trials. When this was added to an analysis of just critical trials, there was no effect of prime, repetition, or the interaction (*p*’s > .25).

## GENERAL DISCUSSION

This project provided the first test of whether reference form choices can be primed in the abstract. Our findings revealed two major results. First, the results from Experiments 1 and 2 provided the first evidence that speakers tend to mimic the referential forms they have recently heard. Participants produced more pronouns when they heard the experimenter use pronouns, and more names when they heard the experimenter use names. But second, this effect was limited: it was small, and did not respond to the most recently-heard trial: in Experiment 1 it did not change in the second block when the prime type changed, and in Experiment 3 there was no evidence for trial-by-trial priming. In fact, the effect seems more like a broad situational adjustment than a flexible modulation of forms based on the most activated linguistic structure.

The effect of priming thus answers our first question, and shows that discourse status does not have a uniform effect on referential choice. Instead, recent experience can modulate the rate of using pronouns vs. names at an abstract level, in particular experience at the start of the current interaction. Critically we showed that this mimcry occurs independently of any effect the prime might have on the discourse status of the primed referent. Both rational and selectional models suggest that form choices are driven by variation in the discourse status of the referent, whether that status is defined as the topicality status of a referent (Chafe, 1976; Grosz et al., 1995; Grosz & Sidner, 1986), its predictability (Arnold, 1998; Kehler et al., 2008; Kehler & Rohde, 2013, 2019; Orita et al., 2015; Tily & Piantadosi, 2009), or attention paid to discourse entities (Brennan, 1995; Gleitman et al., 2007; Myachykov et al., 2012; Tomlin, 1997; Tomlin & Myachykov, 2019), or some combination of these things. The use of a pronoun can make a referent seem more topical/salient (Kameyama, 1996), raising the possibility that discourse status can be confounded with priming, as in many corpus studies. But in all experiments, each trial started a new story, and in Experiment 1, consecutive experimenter and participant trials always used different characters. For example if the experimenter says “he” for Will, then on the next trial if the participant uses “she” to refer to Ana, the experimenter’s pronoun cannot have impacted the perceived topicality/salience of Ana. In Experiments 2 and 3 there was no increase in pronoun use for the trials where the characters were the same as in the priming trial, confirming that discourse status is re-set at the start of a new story.

Our second question concerned the specificity of the primed information. Experiment 1 tested whether priming occurred only when the target referring expression and antecedent were in the same structural relationship as in the prime. However, we found no difference between the same- and different-structure trials. This shows that production priming does not involve the very specific activation of a pronoun-antecedent relation, e.g., “pronoun refers to prior subject”, which contrasts with exposure effects on comprehension (Johnson & Arnold, 2023; Ye & Arnold, 2023). This means that our mimicry effect is likely more general. This could involve an activation of the general class of pronouns as a referring device. It’s even possible that pronouns signal the use of reduced referential expressions in general, or the use of cohesive language in general (Hwang, 2023). Future work is needed to distinguish these possibilities.

One open question is whether priming activates specifically the lexical items “he” and “she” rather than “anaphoric pronouns” as a general class. Experiment 1 always presented primes and targets where the gender of the main character was different. That meant that, for example, if the target story presented an option to use “Liz” or “she”, the prior pronoun prime used “he”. The fact that we still saw priming effects might seem to suggest that priming is not specific to the lexical item. However, we also saw in all three experiments that the priming effect was long-lasting. Therefore it is impossible to rule out the possibility that priming occurred because “she” was encountered earlier.

Our third question concerned the longevity of priming. Results from all three experiments suggest that priming effects are not limited to the immediately following trial. Instead, primes encountered at the start of the session have a lasting effect on the entire task. In Experiment 1, participants followed the prime pattern for the first block, and persisted in using it throughout the second block. This suggests that the priming effect is not only driven by recently encountered referential forms. If it were, we should have seen the effect at least cancel out in the second block. Instead, it seems that the beginning of the experiment provides critical learning about what type of speech is expected during the session. Once participants have encoded the expected pattern, they stick with it even when the experimenter’s production patterns change in the second block. This may be due to lower attention to the experiment in the second block. Alternatively it may indicate that the onset of a new situation (the experimental task) offers a particularly strong learning environment. Participants may specifically track input at the start of an event to learn about the expected speech patterns. If so, situation-based priming effects may be closely related to speech variation across genres (Travis, 2007).

In sum, our findings show that pronoun priming does occur, in that hearing pronouns in one story increases the chance of using pronouns for another referent in a different story. However, this effect is not highly specific. It doesn’t require a similar reference-antecedent relation. It also isn’t limited to the very next referring event, and seems to persist throughout an experimental session. In sum, the so-called “priming” effects we observed do not appear to reflect the automatic activation of a specific linguistic form. Instead, our exposure effects are more diffuse, perhaps reflecting a general activation of pronouns as a class, a broader adjustment to the style of one’s interlocutor or a conceptualization of how to do the task.

One theoretical implication for the lack of a trial-by-trial priming effect is that it speaks against a role for production facilitation in the decision of whether to use a pronoun or more explicit expression. Rational models suggest that production choices are in part driven by efficiency, which has generally been interpreted in terms of the ease of production (e.g., Tily & Piantadosi, 2009). Priming should make it easier to produce matching forms, and indeed there is evidence that production is speeded following exposure to the same form (Jescheniak & Schriefers, 1999). On this model we would expect effects of trial-by-trial priming, which would signal facilitation from the previous trial. However, we saw no such effects in Experiment 3. This is consistent with other evidence against a role for efficiency. For example, planning facilitation is unrelated to pronoun use, and difficulty with speech production (which could be ameliorated by using a pronoun) tends to correlate with the use of explicit forms, and a lower use of pronouns (see Arnold & Zerkle, 2019 for discussion). Thus, our findings argue against the piece of rational models (efficiency) that is distinct from other theoretical approaches.

What do priming effects say about selectional theories (aka salience/accessibility approaches)? These theories suggest that pronouns are appropriate in some contexts due to their pragmatic specialization (e.g., Ariel, 1990; Gundel et al., 1993). Essentially, this view suggests that pronouns are sometimes appropriate because language conventions dictate that they are, and that this can facilitate comprehension. Work in this tradition has identified linguistic contexts such as prior subject (Brennan, 1995) or topicality (Givón, 1983) as conditions that promote pronoun use. Our finding that people did mimic the referential forms of their interlocutor suggests that the impact of the discourse context cannot be totally fixed, and that people modulate their productions in line with their interlocutor’s behavior. This priming effect broadens the selectional view that the discourse context determines appropriate referential forms.

A more general theoretical implication of our findings is that modeling the production of reference should not be limited to the question of how speakers can get listeners to identify the referent, which has been the focus of almost all psycholinguistic work on reference production. Questions about when speakers use pronouns, or even when speakers modify descriptions (e.g., the hat vs. the red hat) are framed mostly in terms of the comprehender’s ability to identify the referent (e.g., see Davies & Arnold, 2019, for a review). If referential identification is the primary problem, then the safest thing for any speaker would be to use highly specific forms all the time, e.g., “the person in the story I just mentioned whose name is Matt.” So why would a speaker ever use an ambiguous form like a pronoun? Rational models suggest that it makes life easy for the speaker (e.g., Orita et al., 2015; Tily & Piantadosi, 2009), but we have seen little evidence to suggest that efficiency modulates speakers’ choices (see also Arnold & Zerkle, 2019).

An alternate view is that pronouns are used because they signal connectivity in the discourse. Arnold and Zerkle (2019; see also Arnold & Nozari, 2017; Hwang, 2023) suggest that this is the communicative function of pronouns and other reduced forms—they connect one proposition with an earlier one (cf. Levelt, 1989). Thus, if the connection between two events is a part of the message they want to communicate, they need to find a way to signal that, and pronouns offer a way to do it. This view suggests that production models must incorporate a representation of event connectivity as a part of the discourse representation. That is, connectivity is part of the “meaning” the speaker communicates. If the connection between events is part of the message, its role may be more or less important. When connectivity as a goal is activated, speakers are more likely to use cohesion devices like pronouns, acoustic reduction, or connector words. When connectivity is not critical, the lack of connection can be signaled by descriptions or names.

Some of the variation in connectivity comes from the discourse itself. For example, in “Liz insulted Ana so she cried”, the tight causal relation between the events is critical for understanding what happened, and speakers may highlight that connectivity with pronouns or by using connector words like “so” or “and”. More generally, when speakers tell stories, they want their addresses to engage with the relation between utterances. But in a series of instructions about how to land a plane, for example, clarity is more important and connection as a goal may be downgraded. The goal of connectivity may be modulated by other factors. Arnold and Nozari (2017) found that speakers used pronouns and zeros more often when the timing of observed visual input required them to plan the description of one event while producing the prior event. They hypothesized that this helped create the sense of connection even when it wasn’t inherent in the actions observed.

Critically, the role of connectivity per se goes beyond the idea that reference form is just determined by the goal of helping the comprehender identify the right referent. This is of course one part of communication, but pronoun use may play an additional function—namely to signal cohesion. This idea is consistent with selectional models, but adds a new criterion for choosing pronouns.

On this view, our current findings may reflect the activation of connectivity as a goal. When the experimenter uses pronouns, it may signal that connectivity is important for the current discourse task. Participants could approach our task in one of two ways: they could see it as a “describe-the-picture” task, where each panel in the input is considered more or less separately. Or they could see it as a “tell the story” task, focusing on the relation between the two panels. Hearing pronouns early in the trial may guide them to take a narrative approach. This is akin to thinking of our observed priming effect as a genre effect.

Another way of characterizing the connectivity proposal is as a stylistic adjustment (perhaps due to genre or other contextual pressures), for example “use reduced forms” or “use a connected discourse style”, as opposed to activation of specific linguistic structures (e.g., the class of pronouns). Once participants hear the experimenter produce connected stories, they activate the goal of producing a connected speech style, which could in theory encourage the use of pronouns, acoustic reduction, and other markers of cohesion. If pronoun priming is a broad stylistic shift, it may be more similar to acoustic/phonetic or sociolinguistic coordination (e.g., Bell, 1984; Ostrand & Chodroff, 2021). This adjustment may be particularly potent for contexts where one person is seen to be the expert, as in our study where participants did the task with an experimenter, or the degree to which participants feel socially connected with their addressee (e.g., Balcetis & Dale, 2005; Hwang & Chun, 2018).

Given that we used a dialogue setting, another way of conceptualizing our priming effect is as speaker-addressee coordination. Interlocutors tend to converge on shared naming conventions (e.g., Brennan & Clark, 1996; Yoon & Brown-Schmidt, 2014) and syntactic structures (Branigan et al., 2000; Ostrand & Ferreira, 2019). In Pickering and Garrod’s (2004) interactive alignment model they propose that this coordination often reflects automatic alignment of the use of similar linguistic structures amongst interlocutors. Yet we still do not know enough about reference form priming to say whether it involves automatic alignment processes.

In conclusion, the current study has provided the first evidence that indeed it is possible to prime the choice between pronouns and names during reference production. This effect occurred across distinct stories in an experimental context and cannot be explained by changes to the discourse status of the referent. These findings constrain current theories on reference production, and the long-lasting nature of this effect argues against the idea that production efficiency affects reference form choices, one of the key tenets of rational models. These findings raise questions about exactly which types of structures are activated and whether this effect is similar to other examples of linguistic priming and alignment, but this study also provides an experimental paradigm that can be leveraged to programmatically test the nature of referential form priming.

## ACKNOWLEDGMENTS

I am grateful to Gabrielle Garner, Ranjani Venkatesh, Caitlin Thompson, Morgan Davis; Zachary Vig, A’sjei Scott, and Jane Xing for their work on this project. The faces for Liz and Will were drawn by Darith Klibanow and are copyrighted to Jennifer Arnold 2020. The faces for Ana and Matt were drawn by Eri Kakoki and are copyrighted to Jennifer Arnold 2022. The full-body images were created using resources from Freepik.com.

## FUNDING INFORMATION

This research was supported by NSF grant 1917840 to J. Arnold.

## DATA AVAILABILITY STATEMENT

Supplementary materials (data, stimuli, and SAS models) are available at https://osf.io/2nyrb/.

## Notes

^1^ Gundel et al. (1993) instead propose that referential forms are implicational, such that a speaker might use a pronoun for a highly focused referent, but a pronoun is not required, and a lower-ranked expression could also be used. This approach is less consistent with a selectional mechanism but it also doesn’t provide an alternative mechanism.^2^ We define a native speaker as someone who began learning English before age 6.^3^ Model includes random slopes by subjects for Current prime, One/Two char, and Current * One/Two char, and by items for First-block prime, Current prime, Structure, One/Two char, One/Two char * First-block prime, One/Two char * Current prime, First-block prime * Current prime, One/Two char * Pronoun prime * First-block prime, Structure * First-blockprime, Structure * Current prime, Structure * One/Two char.^4^ This model includes random intercepts for subjects and items, and a random slope for Post-priming vs. Baseline by subject and for Pronoun prime vs. Name prime by items.^5^ This is a modified version of the practice item in Experiment 1, where only Ana danced.^6^ Due to an error, the first six participants (3 on each list) did not see the practice items, and instead the baseline trials were presented as practice items as in Experiment 2. Seeing the practice items may have increased facility with the task, in that overall there were fewer pronouns produced for these first six participants (0% on baseline; 8% on critical items) than the other participants (2% on baseline, 24% on critical items). However, excluding these participants had no effect on the statistical patterns.^7^ This model includes random intercepts for subject and item, and a random slope for Post-priming vs. Baseline by subject.^8^ This model includes random intercepts for subject and item, and a random slope for Pronoun vs. Name prime by both subject and item.
